# First Case of the Treatment of Massive Tricuspid Regurgitation With the CroíValve DUO Coaptation Valve in a Patient With a Right Ventricular Pacemaker Lead

**DOI:** 10.1016/j.shj.2024.100329

**Published:** 2024-06-25

**Authors:** Ewa Peszek-Przybyła, Marek Jędrzejek, Grzegorz Smolka, Martin J. Quinn, Piotr Pysz, Wojtek Wojakowski

**Affiliations:** a Division of Cardiology and Structural Heart Diseases, Medical University of Silesia, Katowice, Poland; b CMO of CroíValve and St. Vincents University Hospital, Dublin 4, Ireland

**Keywords:** DUO, Pacemaker lead, Transcatheter coaptation system, Transcatheter tricuspid valve intervention, Tricuspid regurgitation

## Abstract

**Background:**

Tricuspid regurgitation (TR) is a common valvular disorder with limited treatment options. It occurs when tricuspid leaflet closure is prevented by dilation of the right heart or in patients with cardiac implantable rhythm devices when the transvalvular lead impedes proper closure of the valve. The management of these patients can be complicated. The removal of the lead often does not improve the TR, and surgical repair is usually not possible because of comorbidities. A number of percutaneous TR repair and replacement devices have been developed; however, the presence of the right ventricular lead can prevent the delivery of these devices, or the device may displace the pacemaker lead. We report the first implant of the CroíValve DUO Transcatheter Tricuspid Coaptation Valve System (Dublin, Ireland) in a patient with massive TR and a right ventricular lead.

**Methods:**

The patient was not a fit for surgical treatment and underwent transcatheter treatment following compassionate use approval. The procedure was performed under general anesthetic with echo and X-ray guidance. The device was delivered through the right internal jugular vein.

**Results:**

The device was implanted successfully, and the TR was reduced from massive to mild at 90-day follow-up. The patient’s quality of life improved significantly with an improvement in 6-minute walk test (382 m at baseline to 467 m at follow-up), the New York Heart Association classification (III at baseline to I at follow-up), and the Kansas City Cardiomyopathy Questionnaire (baseline score 43 increased to 60). The efficacy and clinical improvement have been stable over the past 90 days of follow-up, and the patient has not suffered any adverse events.

**Conclusions:**

This is the first implantation of the CroíValve DUO Coaptation Valve System in a patient with a pacemaker lead. In these patients, this device may offer advantages over other current transcatheter approaches.

## Introduction

Tricuspid regurgitation (TR) is a common valvular disorder with a poor prognosis when left untreated.^1^ TR occurs when dilation of the right heart pulls the tricuspid leaflets apart due to the increase in size of the right atrium or ventricle or due to pulmonary hypertension.^2^ TR can also occur when the tricuspid leaflets are damaged or their movement is reduced due to the presence of pacemaker or implantable cardioverter defibrillator leads. Lead or cardiac implantable electronic device (CIED)-related TR is common, and new or worsening of existing TR has been reported in between 10% and 39% of patients after implantation of a transtricuspid pacing or implantable cardioverter defibrillator lead.^3^ Removal and repositioning of the CIED lead often fails to improve the TR, and the majority of patients with CIED-related TR are not candidates for surgical repositioning of the lead and repair of the tricuspid valve due to comorbidities.^4^^,^^5^ Medical therapy with diuretics is often the only treatment available to these patients. Recently, a number of minimally invasive tricuspid valve therapies have been developed, which may offer an alternative to surgical intervention.^6^ We describe the successful treatment of an 81-year-old patient with massive TR, related to a transvalvular pacemaker lead, with the CroíValve DUO Coaptation Valve (Dublin, Ireland).

## Material and Methods

**Figure 1:**
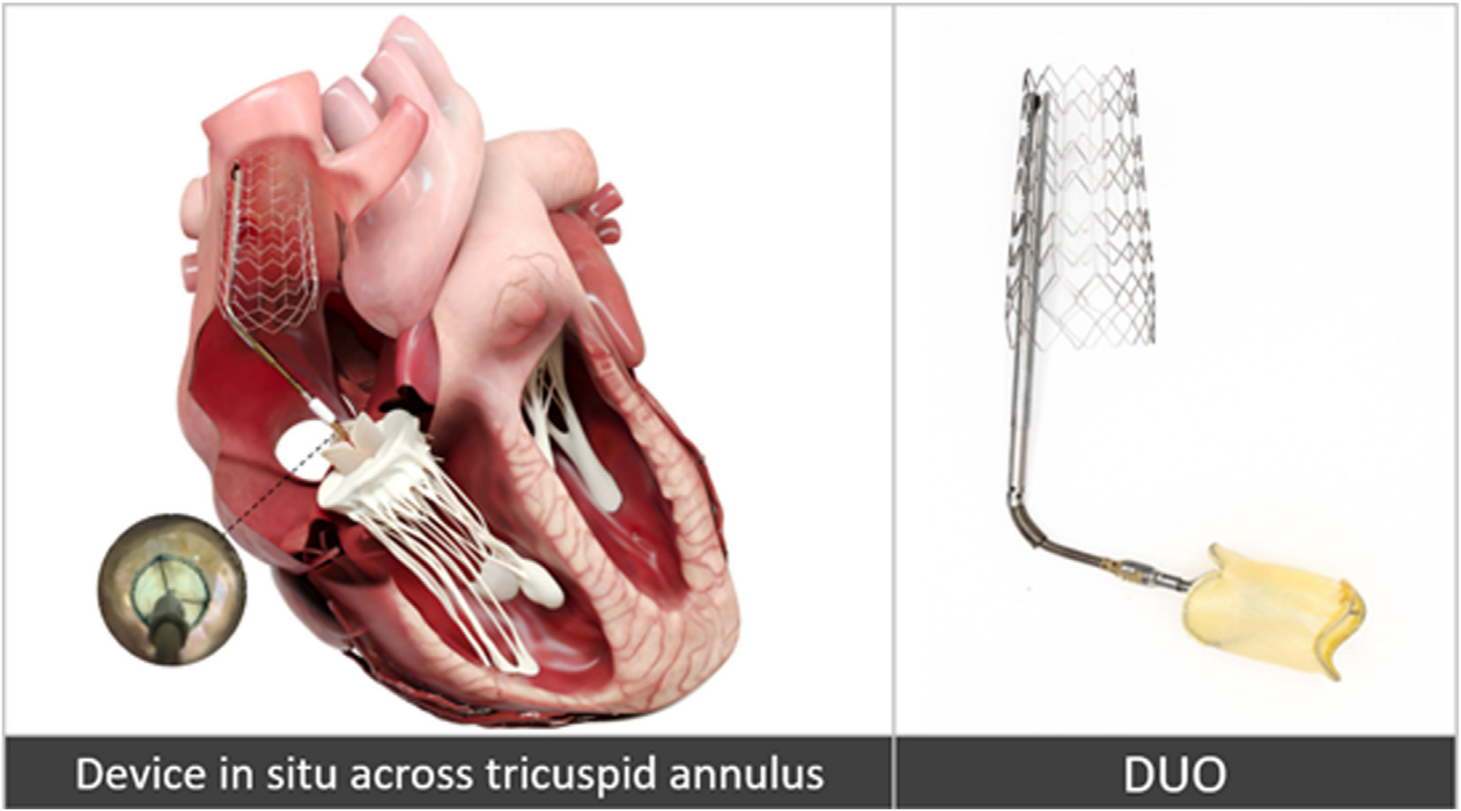
**The DUO transcatheter tricuspid coaptation valve system**.

The DUO Transcatheter Tricuspid Coaptation Valve System is a novel device specifically designed for the tricuspid valve. It consists of a coaptation valve, an adjustable catheter system, and a superior vena cava (SVC) stent (Figure 1). The coaptation valve is positioned between the tricuspid leaflets to fill the regurgitant orifice. The native tricuspid leaflets coapt against the outer cylindrical skirt and thus reduce regurgitation. A valve within the coaptation element allows diastolic flow through the device to facilitate normal hemodynamics. A support catheter suspends the coaptation valve across the tricuspid annulus and is fixed to a self-expanding stent positioned in the SVC. The anchoring approach allows undisturbed function of the right heart, thus avoiding any annular contact and potential disturbance of the conduction system. It also allows the native leaflets to optimize the position of the coaptation valve within the regurgitant orifice, avoiding the need for precision positioning. The stent size is chosen based on preprocedural computed tomography (CT) measurements of the perimeter-derived SVC diameter to ensure appropriate oversizing. The catheter system is adjustable in all planes to accommodate the wide variation in anatomy seen in this patient cohort. The system is crimped into a 22 F delivery sheath and delivered through the right internal jugular vein.

### The Patient

**Figure 2:**
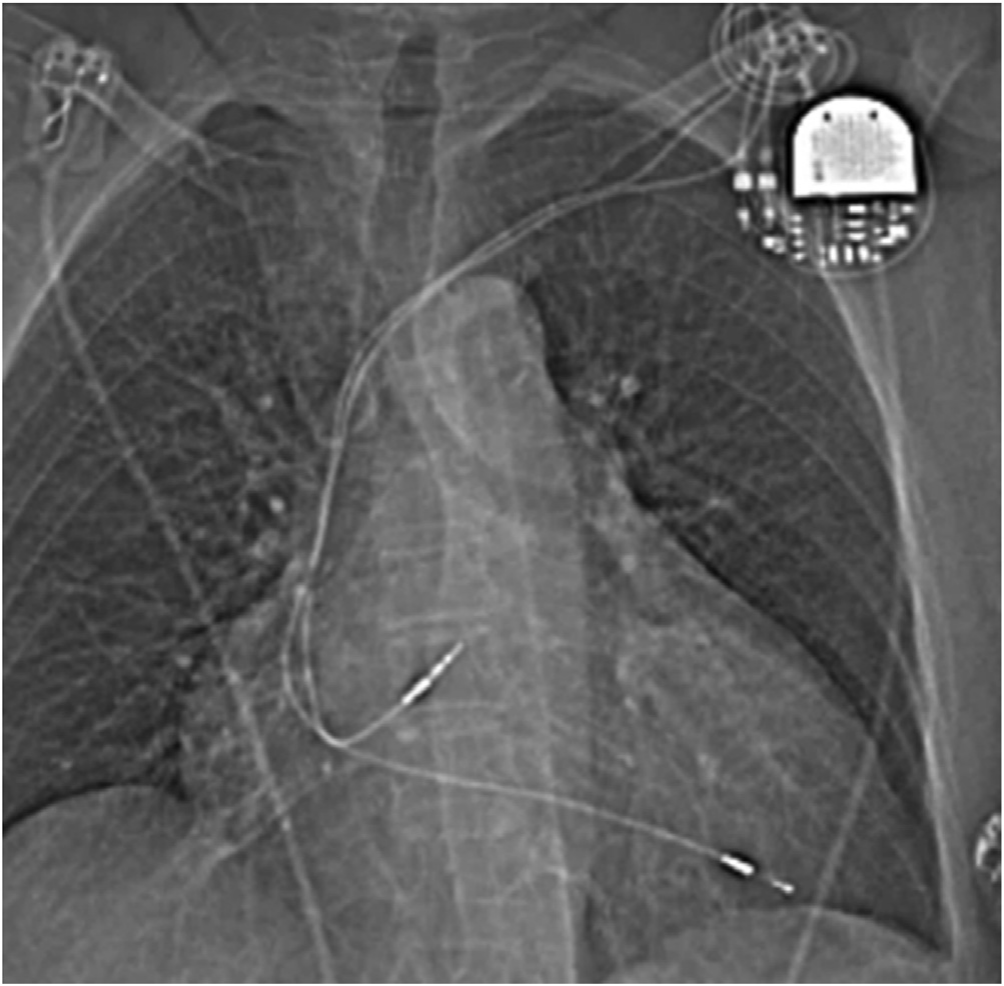
**Patient X-ray showing the presence of the leads and pacemaker**.

**Figure 3:**
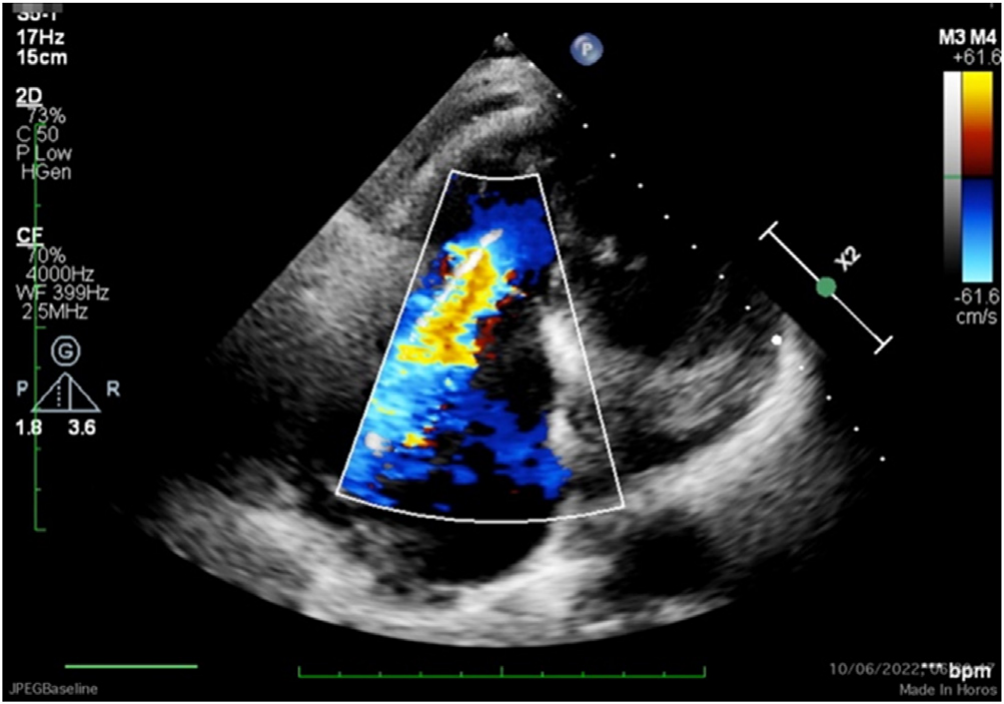
**Baseline transthoracic echocardiography images demonstrating the broad vena contracta (apical RV-focused 4 chamber) view**. Abbreviation: 2D, 2-dimensional; bpm, beats per minute; CF, color flow; RV, right ventricle.

The patient was an 81-year-old female with massive TR related to a transvalvular pacemaker lead. The patient had limiting dyspnea and an associated poor quality of life. The dual-chamber pacemaker had been implanted in December 2005 for sick sinus syndrome. She is pacemaker-dependent. The lead crossing the tricuspid annulus was central and not adherent to the native leaflets (Figure 2). The patient was significantly symptomatic despite medical therapy, and the local heart team determined the patient was too high-risk for surgery. Furthermore, the patient was not suitable for other available transcatheter TR devices due to the size of the leaflet coaptation gap (11 mm coaptation gap in the proximity of the lead). The patient had massive TR at baseline with a large regurgitant orifice (3-dimensional vena contracta area 3.28 cm^2^) and a vena contracta of 15 mm (Figure 3). The patient was fully informed about the procedure and consented to device implantation. Independent core lab adjudication of the degree of TR was performed.

### Procedural Planning

**Figure 4:**
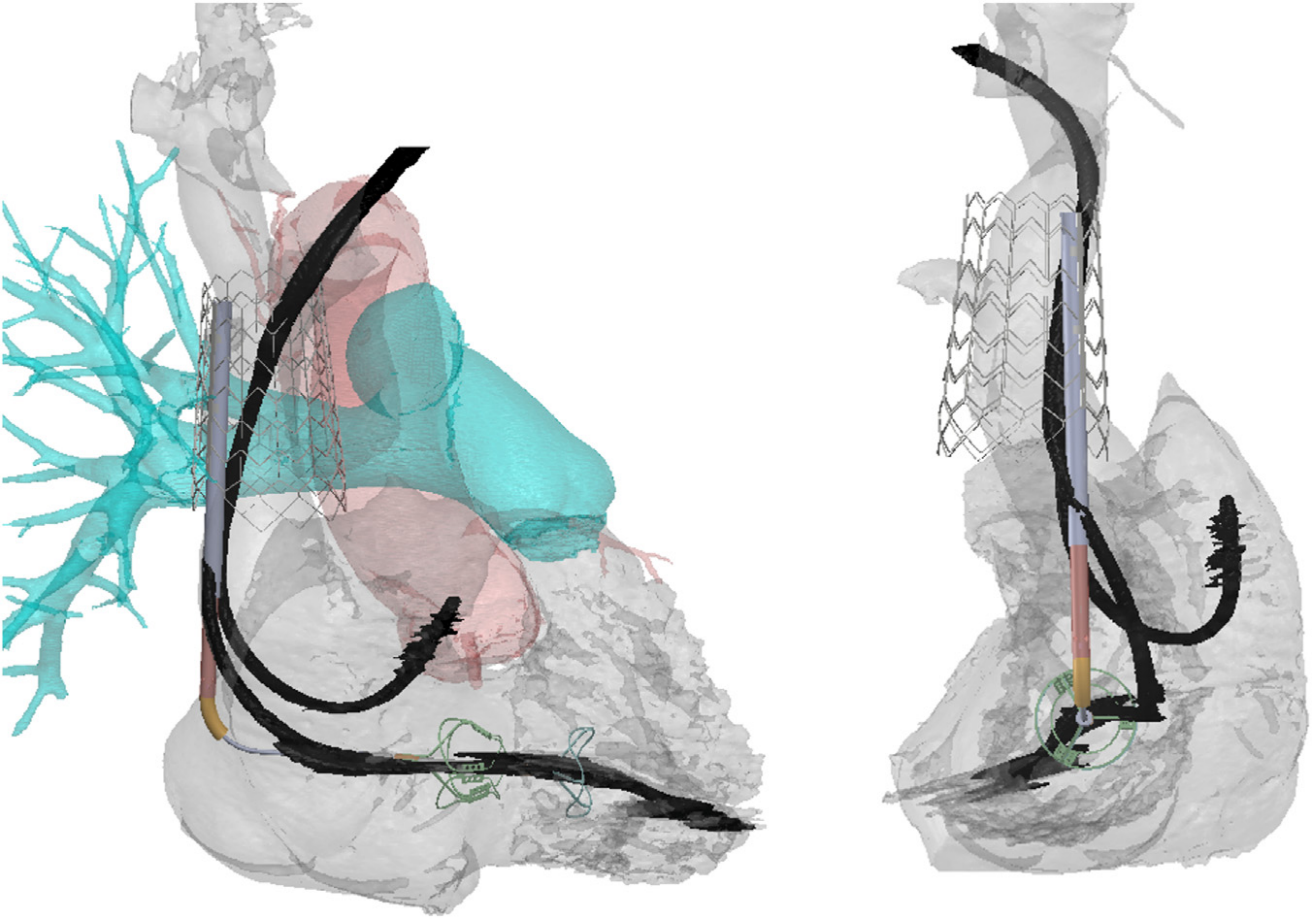
**Patient CT images showing planned fitment and lead position.** The easy-to-identify landing zone for superior vena cava stent is the lower aspect of the right pulmonary artery. Abbreviation: CT, computed tomography.

A cardiac-gated CT was performed for preprocedural planning. The CT was used to measure the diameter of the SVC at multiple levels in order to select the correct SVC stent size. Based on the measurements for this patient, a large stent was selected. In addition, the CT enabled preprocedural planning to assess device fitment and positioning and the possible interaction of the device with the leads (Figure 4).

The procedure was performed under general anesthesia using X-ray and echo guidance in the cardiac catheterization laboratory. Right internal jugular access was obtained under ultrasound guidance. Right heart and SVC anatomy were defined by contrast injection, and an SVC and right atrial road map were stored. This was used to guide device and stent positioning. The right internal jugular access was preclosed using 2 ProGlide devices. The internal jugular access was sequentially dilated to allow the delivery of a 26 F Gore sheath. Heparin was administered to achieve an activated clotting time > 300 ​seconds. The DUO device was delivered into the right atrium through the 26 F sheath. The sheaths were withdrawn, and the coaptation valve self-expanded in the right atrium. The support catheter (which is deflectable and extendable) was flexed to deliver the DUO across the annulus. Once across the annulus, the self-expanding stent was delivered in the SVC. Post-stent delivery, the position of the DUO was adjusted to sit toward the center of coaptation to maximize efficacy and so that the native leaflets co-apt at the midpoint of the coaptation valve at the start of systole. This ensures that there is sufficient length in the skirt to accommodate tricuspid annular plane systolic excursion. When the optimal position was achieved, the support catheter position was locked to the stent, and the delivery system and sheath were removed from the patient. There were no procedural complications.

## Results

**Figure 5:**
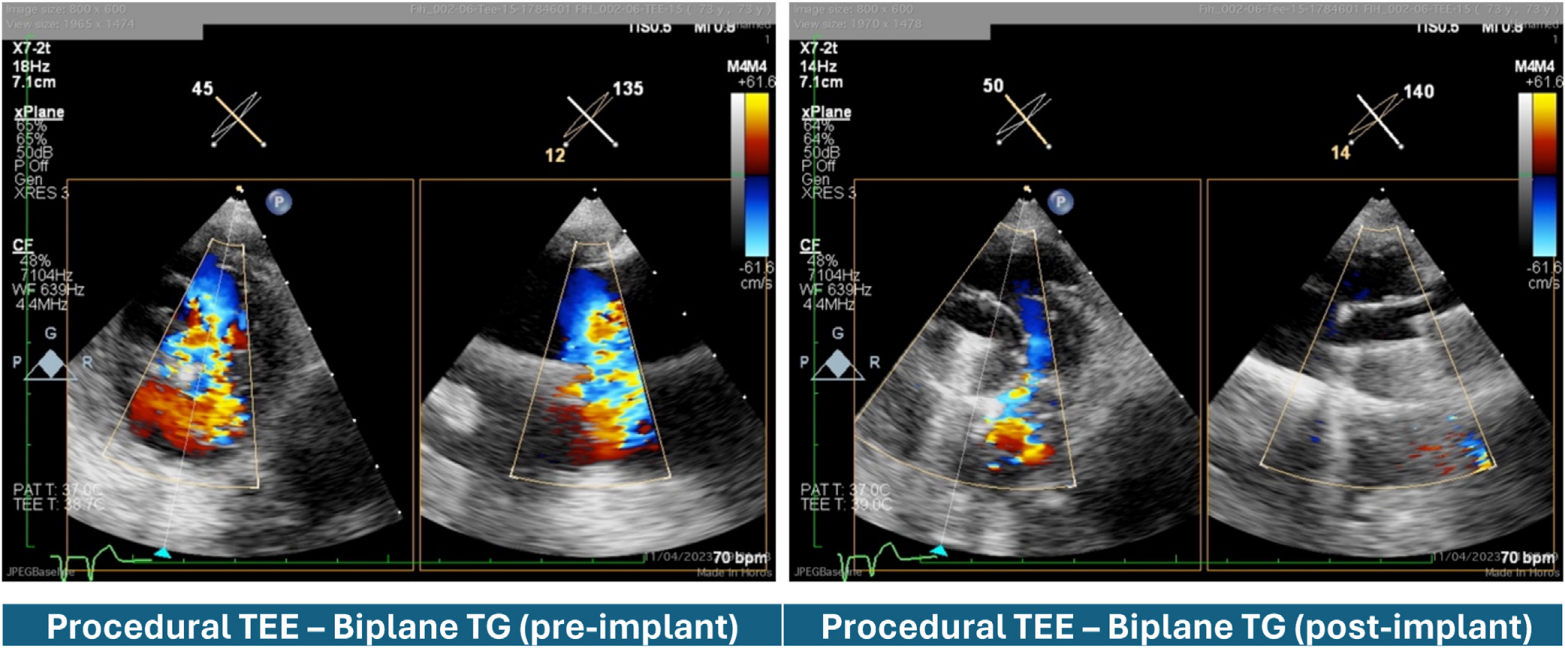
**Biplane procedural TEE views.** On the left transgastric short and long axis view of baseline TR demonstrating the broad central TR jet with the central pacemaker lead. On the right, a similar transgastric bi-plane view after device implantation demonstrating the coaptation valve filling the majority of the regurgitant orifice with mild to moderate residual TR in the anteroseptal commissure and deflection of the lead anteriorly. Abbreviations: 2D, 2-dimensional; bpm, beats per minute; CF, color flow; TEE, transesophageal echocardiography; TG, transgastric; TR, tricuspid regurgitation.

The DUO device was inserted without complications. The TR was reduced from massive to mild and to moderate. The patient remained hemodynamically stable throughout the procedure. The device delivery time was short at 47 ​minutes. The DUO device filled the majority of the large regurgitant orifice and deflected the pacemaker lead anteriorly and toward the septum (Figure 5). The patient recovered well postprocedure and was discharged 5 days postprocedure.

**Figure 6:**
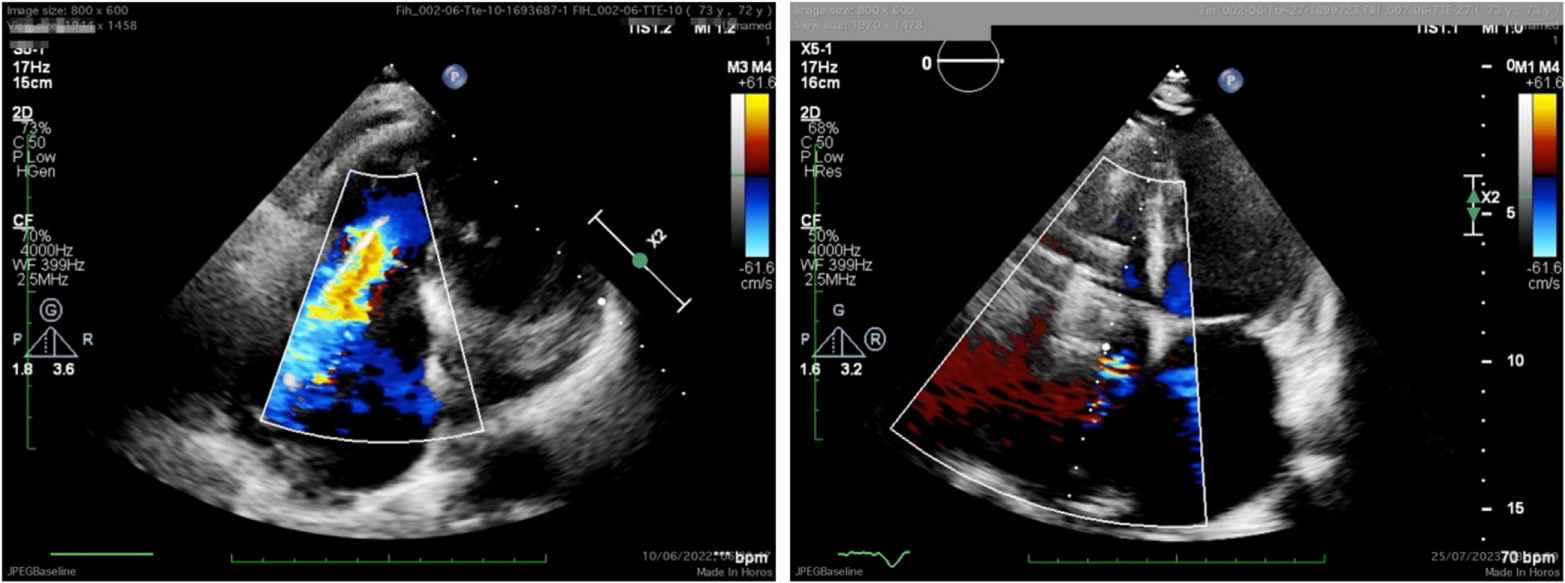
**TR reduction: the left image is the baseline modified 4C view demonstrating the broad jet of TR. The right image is the 90-day follow-up 4C view demonstrating stable mild residual TR in the AS commissure.** Abbreviation: 2D, 2-dimensional; AS, antero-septal; bpm, beats per minute; CF, color flow; TR, tricuspid regurgitation.

**Figure 7:**
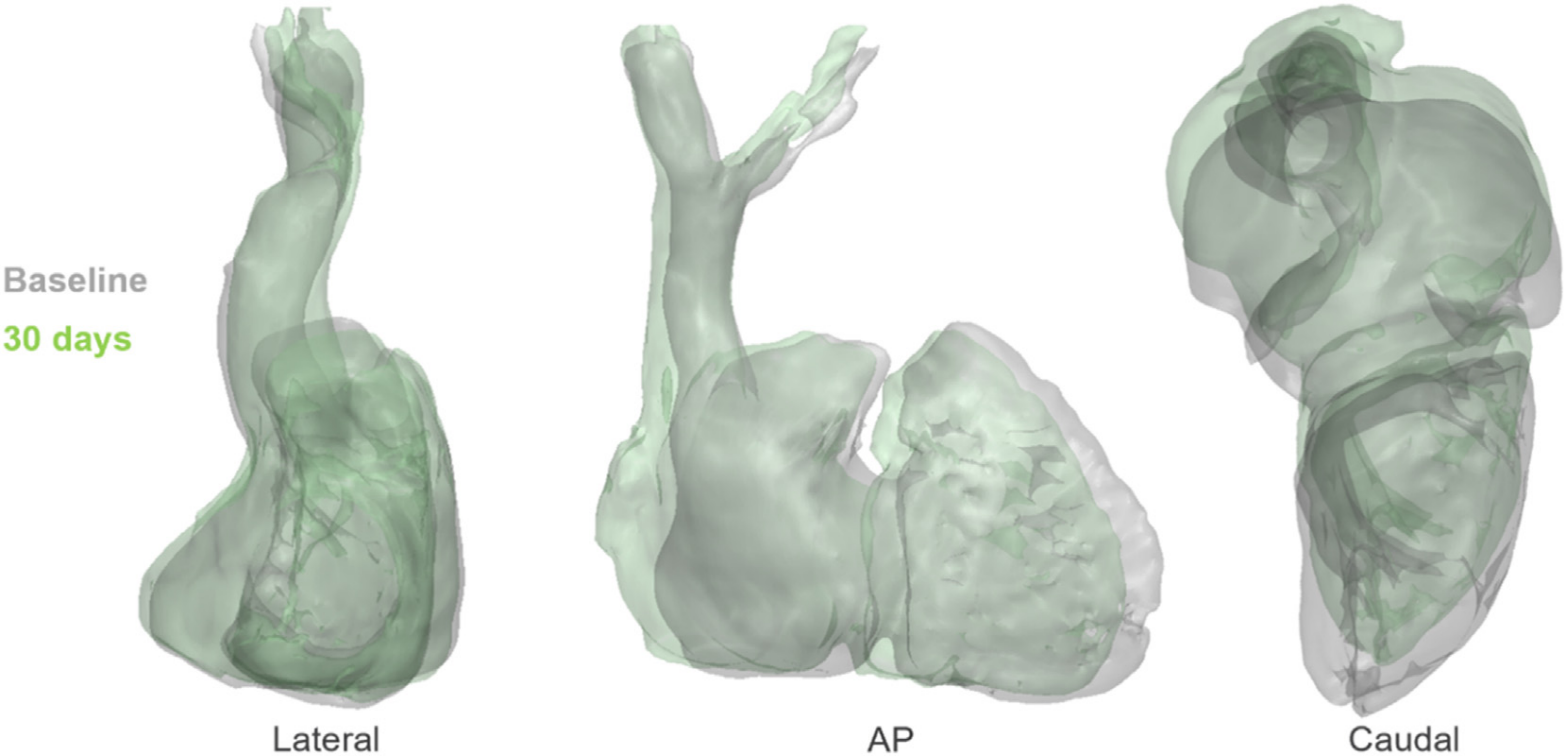
**Overlay of baseline (gray) and 30-day (green) CTs to demonstrate the reduction in right-sided volumes.** RVEDV was reduced by 34%. RV stroke volume measured by the difference between systolic and diastolic volumes was reduced by 33%. Abbreviations: AP, anterior-posterior; CT, computed tomography; RV, right ventricle; RVEDV, right ventricular end diastolic volume.

Follow-up transthoracic echocardiography at 30 and 90 days confirmed stable device position and efficacy with mild residual TR at the most recent follow-up in the antero-septal commissure around the outside of the DUO skirt (Figure 6). At 30 days postprocedure, the patient underwent repeat cardiac CT, and this demonstrated a 34% reduction in right ventricular (RV) end diastolic volume (Figure 7) and a 33% reduction in RV stroke volume, as assessed by the difference in RV systolic and diastolic volumes. The positive remodeling of the free wall of the right heart did not impact device position, which is dictated by septal wall and stent. The patient’s breathlessness, quality of life, and exercise tolerance all improved significantly postprocedure at the 30-day follow-up, with a reduction in the New York Heart Association (NYHA) functional status from NYHA III at baseline to NYHA I, an improvement in quality of life when assessed via the KCCQ-12 questionnaire from 43 to 62 points, and a 6-minute walk test improvement from 382 to 467 m. These improvements have been sustained through 90-day follow-up and there have been no serious adverse events reported for this patient. Pacemaker function has not been impacted by the device.

## Discussion

The management of significant TR in patients with RV leads can be difficult. The insertion of a transtricuspid lead can result in the development of new TR or the progression of existing TR by one to 2 grades in 16% to 34% of patients after CIED implantation.^3^^,^^7^, ^8^, ^9^, ^10^ The lead can become stuck to the leaflets or the subvalvular apparatus, and adhesion of the lead to the tricuspid valve leaflets has been reported in up to 47% of cases in a postmortem study. Additionally, the lead may prevent the proper closure of the leaflets due to direct pressure, usually on the septal or posterior leaflets. The presence of lead-related TR worsens cardiovascular outcomes.^7^^,^^9^^,^^10^ Often, the only treatment available for these patients is diuretic therapy because they have other comorbidities and are not good candidates for surgical intervention. Less invasive percutaneous removal of the lead can be attempted, but it can be complex and often does not improve the TR. Lead extraction is not without risk, and major complications after lead extraction in the European Lead Extraction ConTRolled (ELECTRa) study occurred in 1.7% of patients.^11^ In addition, lead extraction can result in an increase in TR in 11% of patients.^8^

A number of minimally invasive percutaneous treatments have been developed to address TR. These include edge-to-edge repair with clips, percutaneous orthotopic or heterotopic valves, annular reduction devices, and coaptation enhancement devices.^6^ In the recently reported TRILUMINATE pivotal trial, the edge-to-edge repair TriClip device was used to treat 28 patients with severe TR and RV leads.^12^ The outcome of this cohort of patients with lead-related TR has not been reported separately; however, this device requires complex imaging in order to successfully deliver the clip on the leaflets, and the presence of a pacemaker lead could prevent adequate imaging to ensure correct clip delivery. The successful treatment of patients with RV leads has been reported with the EVOQUE valve^13^, ^14^, ^15^ and Moa et al. have reported on the successful treatment of six patients with CIED-related TR with the LUX valve.^16^ However, implantation of a percutaneous valve has the potential to affect lead function in the long term. In the Valve-in-Valve International Database registry of transtricuspid valve replacement (TTVR), 2 of 28 patients undergoing TTVR in a surgical valve or ring had lead failure in follow-up, and in 1 out of the 28 patients, the lead was displaced.^17^ In addition, percutaneous valve implantation can reduce atrio-ventricular node conduction and increase pacing needs, and anatomical exclusions can limit the use of these devices in patients with very dilated annuli or insufficient height in the right atrium or depth in the RV.

This case demonstrates that the CroíValve DUO provides a viable alternative for these patients. This device is currently being studied in patients with severe or greater symptomatic TR despite medical therapy in the TANDEM I trial (ClinicalTrials.gov: NCT05296148). The DUO Coaptation Valve System can be implanted using minimally invasive percutaneous access through the right internal jugular vein with a short procedure that does not result in hemodynamic instability. The device was positioned adjacent to the pacemaker lead between the leaflets of the tricuspid valve, providing a surface for coaptation of the native leaflets and filling the regurgitant orifice. This approach allows free movement of the lead at the annulus and is thus unlikely to affect pacing function. Unlike percutaneous valves, which trap the lead between the metallic frame and the tricuspid annulus and have the potential for lead displacement or damage in the long term. The lead is trapped behind the stent in the SVC after the DUO implant. Lead entrapment will likely prevent lead extraction when required, e.g., in the setting of lead infection. However, this a relatively low occurrence rate^18^^,^^19^ and has become relatively common practice with TTVR devices.^17^ Additionally, jailing the lead in the region of the SVC where it is endothelialized into the vessel wall may result in a lower likelihood of damage to the lead. The device was implanted without the need for complex imaging. The TR reduction was significant from massive to mild and stable, beyond the 3-month follow-up. Recovery after the procedure was quick, and the patient had a significant improvement in their symptoms with a reduction in NYHA class from III at baseline to I at the 3-month follow-up. In addition, their quality of life improved with a 17-point improvement in the Kansas City Cardiomyopathy Questionnaire-12 score, and they were able to walk an extra 85 meters in the 6-minute walk test.

## Conclusions

This is the first implantation of the DUO Transcatheter Tricuspid Coaptation Valve System in a patient with lead-induced TR. The implant was safe and resulted in a reduction in regurgitation, right heart reverse remodeling, and associated improvements in quality of life. For this type of patient, this device may offer advantages over other current transcatheter approaches.

## Ethics Statement

Reasearch reported has adhered to the relevant ethical guidelines. The study has been approved by the ethics committee and patient provided written informed consent.

## Funding

Financial support for the case was provided by CroíValve.

## Disclosure Statement

W. Wojakowski serves on the advisory board of CroíValve. M. Quinn is a co-founder and shareholder in CroíValve; reports a relationship with CroíValve that includes employment, equity or stocks, and travel reimbursement; and has a patent issued to CroíValve.

The other authors had no conflicts to declare.
